# Hydrogen storage in MOFs: Machine learning for finding a needle in a haystack

**DOI:** 10.1016/j.patter.2021.100305

**Published:** 2021-07-09

**Authors:** Lawson T. Glasby, Peyman Z. Moghadam

**Affiliations:** 1 Department of Chemical and Biological Engineering, The University of Sheffield, Sheffield S1 3JD, UK

## Abstract

In recent years, machine learning (ML) has grown exponentially within the field of structure property predictions in materials science. In this issue of *Patterns*, Ahmed and Siegel scrutinize several redeveloped ML techniques for systematic investigations of over 900,000 metal-organic framework (MOF) structures, taken from 19 databases, to discover new, potentially record-breaking, hydrogen-storage materials.

## Main text

Interest in metal-organic frameworks (MOFs) continues to grow as more and more metal-organic structures are discovered and synthesized: there are currently ca. 100,000 MOFs in the Cambridge Structural Database.^1^ Clearly, this huge number of structures creates tremendous opportunities due to the chemical and physical diversity of MOFs, but it also creates the grand challenge of identifying the top-performing materials for a particular application within reasonable timescales. To address this challenge, the use of ML in conjunction with MOFs has also grown significantly in the past 5 years, from gas adsorption prediction to partial atomic charges, band gap, and other mechanical and chemical property predictions.^2^, ^3^, ^4^, ^5^, ^6^, ^7^ Due to their porous nature, fast kinetics, reversibility, and high gravimetric densities, MOFs have been widely studied for many gas-storage problems, including natural gas and hydrogen. Hydrogen storage is a key enabling technology as hydrogen is considered both a future automotive fuel and a medium for energy storage; however, its application has been limited by hydrogen’s low volumetric density at ambient conditions. Current hydrogen-vehicle designs require storage systems based on high-pressure compression, which are costly and could pose safety issues. Design of novel storage systems that can deliver hydrogen with high energy densities have been a recent focus of many studies.^8^, ^9^, ^10^ In this context, computational approaches enabling fast and accurate predictions for the amount of stored hydrogen can play an instrumental role in the identification of outstanding materials on the computer, prior to laboratory synthesis and testing.

In this issue of *Patterns*, Ahmed and Siegel utilized high-throughput machine learning (ML) models to screen through a staggering set of 918,734 structures—predominantly consisting of hypothetical MOFs—to identify top-performing structures for hydrogen storage.^11^ Importantly, this study assesses the performance of 14 ML algorithms that correlate MOFs’ textural properties with their hydrogen-storage capacities. These ML models were restricted to only seven structural features: single crystal density, pore volume, gravimetric surface area, volumetric surface area, void fraction, largest cavity diameter, and pore limiting diameter. Hydrogen adsorption was set for two operating conditions: an isothermal pressure swing at T = 77 K between 5 and 100 bar, and a temperature/pressure swing between 77 K at 100 bar and 160 K at 5 bar.

An extremely randomized trees algorithm was identified as the top-performing ML model for H_2_ uptake assessment, trained on a set of 24,674 MOFs, predicting usable capacities of 820,039 structures. Additionally, the relative importance of input structural features was quantified, concluding that pore volume for gravimetric capacity and void fraction for volumetric capacity are the most important features for H_2_ uptake predictions.

The article offers an unprecedented 8,282 MOFs with gas adsorption capacities that are predicted to exceed current state-of-the art materials—8,187 for pressure swing operation and the remaining 95 for a temperature/pressure swing system. These ML models are available publicly on the web (https://sorbent-ml.hymarc.org), allowing the user to input structural data to receive rapid predictions of H_2_ uptake capacities in a matter of seconds. The project concludes that the potential candidates typically have low densities (<0.31 g/cm^3^) in addition to high surface area (>5,300 m^2^/g), large void fraction (ca. 0.9), and high pore volumes (>3.3 cm^3^/g), and as a result, materials meeting these criteria are highly recommended targets for synthesis. The authors also performed structural analysis of the 8,282 predominantly hypothetical MOFs to probe whether they match previously synthesized structures to aid the process of materials selection for further laboratory tests and synthesis. This investigation found several examples of successfully synthesized MOFs for a number of top-10 candidates for pressure swing adsorption, which were all hypothetical structures, with similarity to MOF-180, MOF-200, PCN-6X series of MOFs, and NOTT-112. The top 10 materials for the temperature and pressure swing system, were also hypothetical, with no crossover between top 10 pressure swing candidates, and a similarity search of the top candidate returned 40 similar MOFs.

In addition to the contribution of new structures toward the discovery of hydrogen-storage materials, this report has benchmarked the ability of 14 ML algorithms that support the capability of modeling for rapid screening of already-synthesized and future MOFs using minimal input data and computation time. This offers a systematic investigation into how ML workflows impact the ability to predict H_2_ storage in MOFs and also provides power-law relations for determining the minimum training size set for achieving a desired ML accuracy.

This is clearly one of the most extensive investigations to date incorporating an enormous pool of structures in the search for advancements in H_2_-storage technologies. Identifying top-performing candidates from this set of mostly hypothetical structures still remains a challenge as there is no simple approach to gauge the cost and ease of experimental MOF synthesis and potential to scale up manufacturing. The results of and methods used in this article are clear evidence of the forward shift to high-throughput screening via ML techniques. This submission is a key contribution to ML models in the field of MOFs, quickly ushering in the replacement of computationally expensive and inaccessible techniques with new methods and tools available to almost any researcher. The availability of the models and databases contained within this project enables drastic improvements in reproducibility and research speed.

We note that, even if synthesis is possible for the top-performing materials identified by ML methods, the next generation of computational screening approaches must begin to consider inclusion of techno-economic analysis, scale-up potential, and stability under process conditions for all notable materials.
